# CREATIVITY IN LATER LIFE: PERSPECTIVES ON ITS FORMS AND MEANINGS

**DOI:** 10.1093/geroni/igz038.1554

**Published:** 2019-11-08

**Authors:** Carolyn E Adams-Price, Danielle K Nadorff

**Affiliations:** 1 Department of Psychology Mississippi State University, Starkville, Mississippi, United States; 2 Mississippi State University, Starkville, Mississippi, United States

## Abstract

Over the past 15 years, gerontologists have become increasingly interested in identifying activities that increase meaning and well-being in older adults’ lives. One such activity is creative activity. New research suggests that creative activity can improve social connections, well-being and self-esteem, and sometimes cognitive processing. Yet some culturally-specific creative activities in which older people participate are not considered creative using standard psychological paradigms of creativity. This symposium will examine new perspectives on creativity in older adults with emphases on creative meaning across the life-span, and the benefits of new and long-term creative hobbies in healthy and frail older adults. Age differences in artists’ perceptions of their creativity will also be discussed. The first presentation by Helen Kivnick will discuss the overall theme of vital involvement in late life from an Eriksonian perspective and its link to the benefits of creative activities. The second presentation by Kate de Medeiros will discuss the creative benefits of a poetry intervention for older adults with dementia. The third presentation by Monika Ardelt will examine young and older nominated visual artists’ perceptions of their own creativity and the creative process, in the context of creative careers. Finally, Carolyn Adams-Price will describe Glaveanu’s sociocultural model of creativity, which can be used to explain the significance of different levels of creativity in a cultural context, from everyday creativity to genius creativity. Glaveanu’s model has great promise for helping us understand the significance of various creative activities for older adults. Danielle Nadorff will be our discussant.

